# Pseudohypoglycemia in Peripheral Artery Disease: a deceiving discrepancy

**DOI:** 10.51894/001c.165591

**Published:** 2026-07-31

**Authors:** Joseph Ghoubaira, Svetoslav Saev, Bryce Reynolds, Jennifer Delongpre

**Affiliations:** 1 Trinity Health Muskegon

## 78

### Introduction

Pseudohypoglycemia, as opposed to hypoglycemia, is a condition where glucose measured by the finger stick method appears falsely decreased compared to actual values. This phenomenon is linked to multiple pathologies that interfere with capillary flow or in vitro glycolysis. While not common, clinicians need to be familiar with this phenomenon to avoid unnecessary testing and inappropriate treatments.

### Case presentation

A 58-year-old female with a history of end-stage renal disease on hemodialysis, peripheral vascular disease, a history of transient ischemic attack, insulin-dependent type 2 diabetes mellitus, was admitted to the vascular surgery service for planned amputations of the right 1st and 5th toes secondary to ischemic ulcerations. She underwent her procedure with no complications, with a wound vacuum placed afterwards. Overnight, an overhead rapid response was called for the patient secondary to hyperventilation and mild diaphoresis with a bedside fingerstick glucose of 23. She was given 12.5 g of dextrose with a recheck measuring at 42. Another dose of dextrose was administered, with a recheck at 20 using 3 different glucometers. Two additional doses of dextrose were administered with no improvement. Given clinical stability and unresolved hypoglycemia on capillary blood glucose measurements, a proximal venous basic metabolic panel revealed a blood glucose of 354. The patient was awake and responsive throughout the measurements. Discordance between her point-of-care glucose and venous glucose was thought to be pseudo-hypoglycemia related to her peripheral vascular disease. It was hypothesized that diffuse calcifications in the extremities lead to poor perfusion of the distal limbs, thereby increasing glucose extraction by tissues due to reduced capillary flow and increased transit time. This phenomenon often leads to falsely low peripheral glucose readings. Her initial symptoms of hyperventilation and mild diaphoresis may have been due to her postoperative pain. Subsequent venous draws were performed to ensure stable glucose levels thereafter. The patient remained symptom-free after pain was controlled and was discharged from the hospital after a few days.

### Conclusion

This case represents the unreliability of fingerstick glucose measurements in certain situations. Although hypoglycemia is a clinical diagnosis, awareness of this phenomenon is essential to explain an often-inconsistent clinical presentation.

